# Gedatolisib Combined with Palbociclib and Letrozole in Patients with No Prior Systemic Therapy for Hormone Receptor–Positive, HER2-Negative Advanced Breast Cancer

**DOI:** 10.1158/1078-0432.CCR-25-0992

**Published:** 2025-07-25

**Authors:** Robert Wesolowski, Hope S. Rugo, Jennifer M. Specht, Hyo S. Han, Peter Kabos, Ulka Vaishampayan, Seth A. Wander, Keerthi Gogineni, Alexander Spira, Anne F. Schott, Maysa Abu-Khalaf, Sarah C. Mutka, Samuel Suzuki, Brian Sullivan, Igor Gorbatchevsky, Rachel M. Layman

**Affiliations:** 1 The Ohio State University Comprehensive Cancer Center, Columbus, Ohio.; 2 University of California, San Francisco Comprehensive Cancer Center, San Francisco, California.; 3 Fred Hutchinson Cancer Center, University of Washington, Seattle, Washington.; 4 Moffit Cancer Center, Tampa, Florida.; 5 University of Colorado Hospital, Aurora, Colorado.; 6 Karmanos Cancer Institute, Detroit, Michigan.; 7 Massachusetts General Hospital, Harvard Medical School, Boston, Massachusetts.; 8 Emory University Hospital, Atlanta, Georgia.; 9 Virginia Cancer Specialists Research Institute, Fairfax, Virginia.; 10 University of Michigan, Ann Arbor, Michigan.; 11 Sidney Kimmel Comprehensive Cancer Center at Jefferson Health, Philadelphia, Pennsylvania.; 12 Celcuity, Inc., Minneapolis, Minnesota.; 13 The University of Texas MD Anderson Cancer Center, Houston, Texas.

## Abstract

**Purpose:**

Nonclinical evidence demonstrating that estrogen receptor, cyclin-dependent kinases 4 and 6 (CDK4/6), and PI3K/AKT/mTOR (PAM) pathways cross-promote tumor proliferation in hormone receptor–positive (HR+)/HER2− breast cancer cell lines led to the development of CDK4/6 inhibitors and agents inhibiting single PAM pathway nodes to treat HR+/HER2− advanced breast cancer. Simultaneous blockade of the estrogen receptor, CDK4/6, and PAM pathways may optimize antitumor control in the treatment-naïve advanced breast cancer setting. Gedatolisib, a pan-PI3K/mTOR inhibitor, was evaluated as first-line therapy, combined with standard-of-care palbociclib and letrozole, for patients with HR+/HER2− advanced breast cancer.

**Patients and Methods:**

Treatment-naïve patients from a phase Ib study with HR+/HER2− advanced breast cancer treated with gedatolisib plus palbociclib and letrozole were analyzed. The primary endpoint of the overall study was investigator-assessed objective response. Secondary endpoints included safety, duration of response, progression-free survival (PFS), and overall survival.

**Results:**

Of 41 patients, all had stage IV disease, 93% had measurable disease, 78% had visceral metastases, and 22% had detectable *PIK3CA* mutations. The objective response rate was 79% in patients with evaluable disease (*N* = 33). The median duration of response was 48 months for confirmed responders. The median PFS was 48.4 months, and the median overall survival was 77.3 months. The overall response rate and PFS were comparable in patients with and without *PIK3CA* mutations. Fewer than 10% discontinued treatment due to treatment-related adverse events. The most frequent grade 3/4 adverse events were neutropenia (61%), rash (39%), and oral stomatitis (29%).

**Conclusions:**

Gedatolisib plus palbociclib and letrozole demonstrated preliminary efficacy in patients with no prior systemic therapy for advanced breast cancer. These results warrant further evaluation of gedatolisib added to standard-of-care, first-line therapy for HR+/HER2− advanced breast cancer.

**Translational Relevance**

Gedatolisib, a pan-PI3K/mTOR inhibitor, combined with a cyclin-dependent kinases 4 and 6 inhibitor (palbociclib) and endocrine therapy (letrozole, an aromatase inhibitor), was well tolerated and elicited a 79% objective response rate in first-line patients with hormone receptor–positive, HER2− advanced breast cancer. The median progression-free survival was 48.4 months, and the median overall survival was 77.3 months in first-line patients receiving gedatolisib, palbociclib, and letrozole triplet therapy. Unlike currently available inhibitors of the PI3K/AKT/mTOR pathway, gedatolisib-based triplet therapy achieved comparable clinical responses in patients with and without *PIK3CA* mutations. The multinode inhibition exhibited by gedatolisib may elicit a response in a broader patient population as the mechanism of action does not depend on a specific mutation subtype. These results warrant further evaluation of gedatolisib added to standard-of-care, first-line therapy for hormone receptor–positive, HER2− advanced breast cancer.

## Introduction

Breast cancer is the most common tumor type diagnosed in women in the United States and is the leading cause of cancer death in women globally (Supplementary Table S1; ref. 1). Breast cancer is categorized into subtypes based upon molecular characteristics such as the expression status of the hormone receptors (HR), specifically estrogen receptor (ER) and/or progesterone receptor, and the expression of HER2. The most common molecular subtype is HR+, HER2− breast cancer, which accounts for 74% of newly diagnosed cases (2). For patients with HR+, HER2− metastatic breast cancer, median overall survival (OS) is 4 to 5 years (1). Patients with metastatic breast cancer whose early breast cancer relapsed during or within 12 months of adjuvant endocrine therapy (ET) have a significantly worse prognosis (3).

Current National Comprehensive Cancer Network (NCCN), American Society of Clinical Oncology (ASCO), and European Society for Medical Oncology (ESMO) guidelines recommend ET combined with cyclin-dependent kinases 4 and 6 (CDK4/6) inhibitors as standard-of-care first-line treatment for patients with HR+, HER2− locally advanced (inoperable) or metastatic breast cancer (4). ETs such as selective estrogen receptor degraders (e.g., fulvestrant) and aromatase inhibitors (e.g., letrozole) block tumor cell proliferation by disrupting ER pathway activation. CDK4/6 inhibitors disrupt cell proliferation and tumor growth by blocking the cell-cycle transition from the G_1_ phase to the S-phase. Patients with HR+, HER2− advanced breast cancers treated with CDK4/6 inhibitors plus ET eventually experience disease progression. One key mechanism of resistance to ET plus CDK4/6 inhibitor regimens involves the PI3K/AKT/mTOR (PAM) pathway. Preclinical research has found that cross-talk among the ER, cyclin D1/CDK4/6, and PAM pathways can induce resistance through compensatory mechanisms when only one of these pathways is inhibited (5, 6). Dysregulated PAM pathway signaling induces ER transcriptional activity, whereas ER pathway signaling activates the PAM pathway by the direct binding of ER-α to PI3Kα. Due to the cross-talk between the ER and PAM pathways, inhibition with a PAM inhibitor ultimately leads to increased sensitivity to ET (7, 8). These findings spurred the clinical development of many PAM inhibitors over the past 20 years. To date, four therapies (i.e., alpelisib, everolimus, inavolisib, and capivasertib), each targeting a single node of the PAM pathway, have been approved when given in combination with ET for the treatment of patients with HR+, HER2− breast cancer whose disease progressed on or after ET in the advanced setting. Additionally, inhibition of the PAM pathway has been found to increase cyclin D1 and CDK4/6 activity, which promotes proliferative cell cycling and can either increase sensitivity to CDK4/6 inhibition or restore sensitivity in tumor cells that have become resistant to CDK4/6 inhibition (9). Thus, there is a strong rationale to simultaneously blockade the ET, CDK4/6, and PAM pathways to optimize antitumor control in either the first- or second-line settings. The relevance of blockading all three pathways was confirmed when inavolisib, a PI3Kα inhibitor, was approved by the FDA as first-line treatment for adults with endocrine-resistant HR+, HER2− advanced breast cancer when combined with palbociclib and fulvestrant based on the favorable results of the INAVO120 trial (10).

Gedatolisib is a potent kinase inhibitor that targets all four class I PI3K isoforms and the mTOR complexes mTORC1 and mTORC2. Due to cross-talk between the various PAM nodes, the targeting of multiple PAM nodes by gedatolisib may prevent the development of resistance and thus induce a more comprehensive blockade of the PAM pathway than therapies that only target a single PAM node. A multicenter, open-label, phase Ib study in adult women with HR+, HER2− advanced breast cancer was conducted to evaluate gedatolisib combined with palbociclib and ET in the first-line and later-line settings (11). Promising preliminary efficacy was reported in each setting, reflected in an extended median progression-free survival (mPFS) and a favorable objective response rate (ORR). Of note, the response to the triplet combination was independent of *PIK3CA* mutation status (11).

In this study, we report the efficacy and safety results in a subgroup analysis from the phase Ib study of patients with HR+, HER2− advanced breast cancer who received gedatolisib, palbociclib, and letrozole as their first-line treatment for advanced disease. The current analysis combines patients who participated in either the dose escalation or dose expansion portions of the trial and provides updated data based on a May 29, 2023, cutoff.

## Patients and Methods

### Study design

This multicenter, open-label, phase Ib trial included two dose escalation and four dose expansion arms in which patients with HR+, HER2− advanced breast cancer were enrolled. No randomization occurred. The original results from the dose expansion arms were previously reported (11). This *post hoc* analysis includes all patients with HR+, HER2− advanced breast cancer who had received no prior systemic therapy for advanced breast cancer from the dose escalation and dose expansion portions of the phase Ib study. These patients received the combination of gedatolisib (all patients received a 180 mg dose) administered intravenously once weekly in a 28-day cycle, along with standard-of-care palbociclib (125 mg) once daily for 21 days followed by 7 days off, and once daily letrozole (2.5 mg).

The study was performed in accordance with the Declaration of Helsinki and in compliance with good clinical practice guidelines and applicable laws. The protocol was approved by the institutional review boards at the study sites. Written informed consent before study entry was obtained from each patient or from the patient’s legally authorized representative if the patient was unable to provide consent.

### Patients

The subgroup analyzed consisted of adult (age at least 18 years) female patients with HR+, HER2−, metastatic breast cancer who were eligible to receive a CDK4/6 inhibitor and letrozole, had received no prior systemic therapy in the metastatic setting, and were enrolled in either the dose escalation or dose expansion portion of the study. As eligibility for palbociclib and letrozole therapy required patients to have completed their course of adjuvant ET, if applicable, more than 12 months from the diagnosis of advanced breast cancer, the patients enrolled would generally be considered endocrine-sensitive. Patients were required to have an Eastern Cooperative Oncology Group performance status of 0 to 1 and adequate hepatic, renal, and bone marrow function. Patients were ineligible if they received prior treatment with an mTOR or PI3K inhibitor, had prior hematopoietic stem cell or bone marrow transplantation, or had active, uncontrolled, or symptomatic central nervous system metastases. The protocol mandated that LHRH, GnRH, or equivalent agents to suppress ovarian function be administered to premenopausal or perimenopausal women. Furthermore, patients were required to have adequate glucose control for inclusion, as defined by hemoglobin A_1c_ (HbA1c) <8% and fasting blood glucose ≤126 mg/dL (7.0 mmol/L).

### *PIK3CA* mutation analysis

*PIK3CA* mutational status was determined by analysis of DNA isolated from plasma samples collected before the dose on cycle 1, day 1. Nine *PIK3CA* mutations (C420R, E542K, E545K, E545G, Q546K, M1043I, H1047Y, H1047R, and H1047L) were analyzed using Beads, Emulsions, Amplification, and Magnetics technology (Sysmex Inostics, RRID:SCR_025629). Beads, Emulsions, Amplification, and Magnetics technology performs single-molecule PCRs on magnetic beads in water-in-oil emulsions, as described elsewhere (12, 13). Tumor tissue sequencing for *PIK3CA* mutational status was not performed.

### Efficacy assessments

Objective response status [complete response (CR), partial response (PR), stable disease (SD), progressive disease (PD)] was assessed by the investigator and confirmed by a subsequent radiological scan ≥4 weeks later per Response Evaluation Criteria in Solid Tumors (RECIST) guideline version 1.1. While on study, tumor assessments (CT or MRI) were initially performed every 8 weeks starting from the first day of study drug for at least 18 months or until PD. Twelve months after study enrollment was completed, the protocol was amended to allow tumor assessment to occur every 12 to 16 weeks. Best objective response was defined as the best response across all time points after all tumor assessments were complete for each patient. The ORR, defined as the proportion of patients who achieved a radiologically confirmed PR or CR, was assessed using RECIST version 1.1. The response evaluable population in this *ad hoc* subgroup analysis consisted of patients who had measurable disease, an adequate baseline assessment of disease, and at least one postbaseline measurable assessment of disease.

Secondary efficacy analyses included progression-free survival (PFS) and duration of response (DOR). PFS was determined by the time from the date of the first dose of study drug to the date of the first radiologic documentation of postbaseline disease progression or death due to any cause. DOR was determined from the time of the first documentation of CR or PR to the date of the first documentation of PD or death, whichever occurred first. PFS and DOR were determined using the full analysis set. OS analysis was performed *post hoc* as a separate survival study after the original study was completed and was defined as the time from the start of the study drug to death due to any cause. At the time of the final analysis, alive subjects were censored on the date of the last follow-up in which the subject was known to be alive.

### Safety assessments

All patients were monitored for adverse events (AE) with a treatment-emergent AE (TEAE), defined as an AE occurring from the time of the first dose of study therapy until at least 28 days after the last dose of the study drug. All AEs were coded using MedDRA and graded for severity via the Common Terminology Criteria for Adverse Events version 4.03. Safety laboratory assessments, vital signs, 12-lead electrocardiograms, and Eastern Cooperative Oncology Group performance status were summarized by visit.

### Statistical analysis

All statistical tests were summarized descriptively. Time-to-event data were summarized using Kaplan–Meier methods, and confidence intervals (CI) for median time to event were estimated using the Brookmeyer–Crowley method. Statistical analyses were done with SAS software (version 9.4; RRID:SCR_008567).

### Data availability

The data are available from the corresponding author upon reasonable request.

## Results

### Patients

A total of 41 patients with no prior systemic therapy for advanced breast cancer from the dose escalation cohort (*N* = 11) and from the dose expansion cohort (*N* = 30) were pooled for the analysis (Supplementary Fig. S1). At study end, 32 (78%) patients in the subgroup discontinued treatment. The primary reasons for discontinuation were disease progression or relapse (*N =* 15, 37%), treatment-related AEs (*N =* 4, 10%), AEs of unknown relationship to treatment (*N* = 1, 2%), withdrawal by subject (*N =* 6, 15%), lost to follow-up (*N =* 1, 2%), global deterioration (*N =* 2, 5%), protocol violation (*N =* 2, 5%), and treatment for other malignancy (*N =* 1, 3%). The remaining 9 (22%) of the 41 patients completed the study and continued treatment in an expanded access protocol. As of the last data cutoff on May 29, 2023, five of the nine patients remained enrolled in the expanded access protocol. There was no incidence of death prior to disease progression.

Demographics and baseline characteristics are described in Table 1. No patients received prior systemic therapy for advanced breast cancer. The median age of patients in the study was 54 years. All patients had metastatic breast cancer at enrollment. A total of 38 (93%) patients had measurable baseline disease. The three patients without measurable baseline disease were enrolled in the dose escalation portion of the study, which did not require measurable disease, and were therefore excluded from the response evaluable set due to the absence of target lesions. Patients in this analysis predominantly had three or fewer disease sites involved (85%), and 78% had visceral metastases. ctDNA analysis showed that *PIK3CA* mutations were found in 22% of the total treatment-naïve cohort (Table 1).

**Table 1.** Patient demographic and baseline disease characteristics in the full analysis population with no prior systemic therapy for advanced breast cancer.

​	No prior systemic therapy (*N* = 41)
Age	​
Median years (range)	54 (28–78)
Race, *n* (%)	​
Black or African American	5 (12%)
White	35 (85%)
Other	1 (2%)
TNM current stage, *n* (%)	​
Stage IV	41 (100%)
Number of prior systemic therapies for advanced disease, *n* (%)	​
0	41 (100%)
Prior neoadjuvant chemotherapy, *n* (%)	6 (15%)
Prior adjuvant chemotherapy, *n* (%)	11 (27%)
Prior adjuvant ET, *n* (%)	18 (44%)
Measurable baseline disease, *n* (%)	​
Yes	38 (93%)
No	3 (7%)
ECOG performance status, *n* (%)	​
0	28 (68%)
1	13 (32%)
Visceral metastasis, *n* (%)	​
Yes	32 (78%)
No	9 (22%)
Metastatic site involved, *n* (%)	​
Bone	26 (63%)
Bone only	1 (2%)
Brain	0
Liver	15 (37%)
Lung	7 (17%)
Lymph node	12 (29%)
Pleural effusion	4 (10%)
Skin	1 (2%)
Other	35 (85%)
Number of sites involved, *n* (%)	​
≤3	35 (85%)
≥4	6 (15%)
PIK3CA, *n* (%)	​
Wild-type	31 (76%)
Mutant	9 (22%)
Unknown/missing	1 (2%)

Abbreviations: ECOG, Eastern Cooperative Oncology Group; TNM, tumor–node–metastasis.

### Response

**Figure 1.**
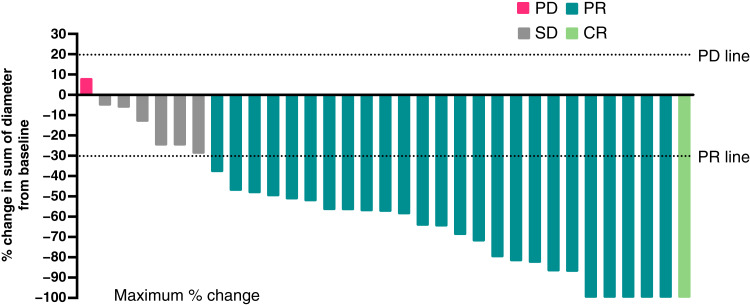
Best overall response and target lesion improvement. Percentage change from baseline in the sum of the diameters of all target lesions at the time of best response. There were five subjects with 100% target lesion responses, but they were classified as PR due to nontarget lesions. Each bar represents a single patient with no prior systemic therapy for advanced breast cancer in the response evaluable set (*N* = 33).

In the treatment-naïve patients who were response evaluable (*N =* 33 out of 41), one (3%) patient achieved a CR, and 25 (76%) patients achieved a PR, yielding an ORR of 79% (Table 2; Fig. 1). Six patients had a 100% reduction in the sum of target lesion diameters. However, five of those patients were classified as PR due to no change in characteristics for nontarget lesions (Fig. 1). Of the six patients total who had SD as their best overall response, three had durable SD greater than 24 weeks. There were three patients in the escalation arm who had measurable nontarget lesions and were not included in the response analysis set due to the absence of target lesions. However, based on the measurement of their nontarget lesions, these three patients would have been categorized as having SD as their best response. The median DOR, which was calculated using the confirmed responders (CR and PR) in the full analysis set, was 47.9 months (95% CI, 24.6 to not reached). The median follow-up time for DOR was 49.1 months (95% CI, 24.3–52.7).

**Table 2.** Response rates to gedatolisib + palbociclib + letrozole in the response evaluable set.

​	Response evaluable WT *PIK3CA*^a^ (*N* = 25)	Response evaluable mut *PIK3CA* (*N* = 7)	Total response evaluable (*N* = 33)
ORR, *n* (%)^b^	20 (80%)	5 (71%)	26 (79%)
Median DOR, months (95% CI)^b^	48 (24, NR)	47 (4, NR)	48 (25, NR)
Best overall response, *n* (%)	​	​	​
CR	1 (4%)	0	1 (3%)
PR	19 (76%)	5 (71%)	25 (76%)
SD	4 (16%)	2 (29%)	6 (18%)^c^
PD	1 (4%)	0	1 (3%)

Abbreviation: NR, not reached.

a *PIK3CA* mutation status was missing for one patient.

b ORR was determined in the response evaluable population (*N* = 33), which corresponded to the patients with baseline target lesions, measurable baseline disease, and at least 1 postbaseline measurement of disease, out of 41 patients in the full analysis set; median DOR was evaluated in confirmed responders in the full analysis population (*N* = 41).

c Three (9%) patients experienced durable SD, defined as SD for >24 weeks.

### Survival

**Figure 2.**
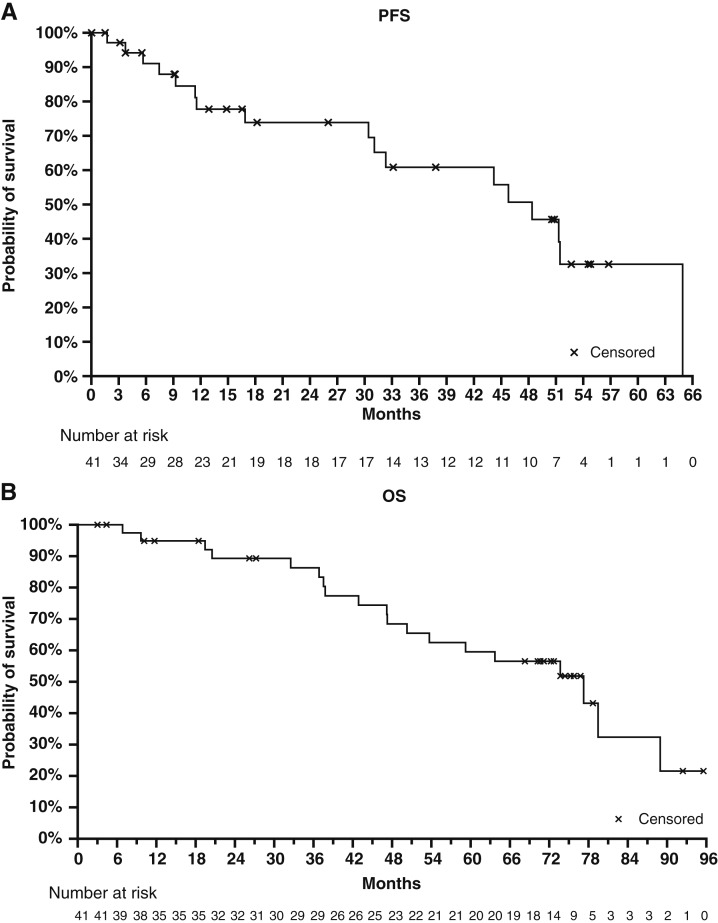
Survival in patients with no prior systemic therapy for advanced breast cancer in the full analysis population. Kaplan–Meier curve determined in the full analysis population of (**A**) PFS and (**B**) OS. PFS was determined by the time from the date of the first dose of study drug to the date of the first radiologic documentation of postbaseline disease progression or death due to any cause. OS was defined as the time from the start of the study drug to death due to any cause.

At the time of data cutoff on May 29, 2023, 15 of 41 patients experienced disease progression, and the median PFS was 48.4 months (95% CI, 30.4 to not reached; Fig. 2A). The estimated 12-month PFS was 78% (95% CI, 59–89; Fig. 2A). The median follow-up duration for PFS was 37.8 months (95% CI, 14.9–54.6), per the reverse Kaplan–Meier method.

At data cutoff among the full analysis set (*N* = 41), there were 19 patients with an event of death and 22 patients censored for OS (Fig. 2B). The *post hoc* analysis of median OS was 77.3 months (95% CI, 50.3–89) over a median follow-up time for survival of 73.7 months (95% CI, 3+ to 95.6+). The estimated 12-month OS was 95% (95% CI, 81–99).

### *PIK3CA* mutation status

Exploratory analyses revealed that ORR and PFS results were comparable in patients with and without *PIK3CA* mutations. In the response-evaluable population whose *PIK3CA* status was confirmed (*N =* 32), the ORR in patients with wild-type *PIK3CA* tumors was 80% (20/25) compared with 71% (5/7) in patients whose tumors had detectable *PIK3CA* mutations. Estimated PFS at 12 months was 79% (95% CI, 56–91) compared with 71% (95% CI, 26–92) in the wild-type and mutated *PIK3CA* populations, respectively.

### Safety

Safety data were summarized using the full analysis set (*N =* 41). All patients reported TEAEs of any grade, which are summarized in Table 3 by order of frequency for all grades. The most frequent grade 3 or 4 TEAEs reported were decreased neutrophil count and/or neutropenia (51%), a known adverse reaction to palbociclib: rash (39%) and stomatitis (29%; Table 3). No prophylactic measures for rash or stomatitis were required in the study protocol. Treatment-emergent serious AEs (TESAE) occurred in 18 (44%) of the 41 patients: 15 (37%) had grade 3 events, and 3 (7%) had grade 4 events, including 2 grade 4 TESAEs in one patient (Table 4). None of the grade 4 TESAEs was assessed as related to study treatment. Four patients (9.8%) discontinued treatment due to an AE deemed probably or possibly related to treatment, and no AEs leading to deaths were considered treatment-related, as assessed by investigators.

**Table 3.** TEAEs (of any cause, occurring in >20% of patients in the safety analysis set; *N* = 41).

Preferred term	Grade 3	Grade 4	All grades
	*N* (%)	*N* (%)	*N* (%)
Patients with any TEAE	34 (83%)	7 (17%)	41 (100%)
Stomatitis^a^	12 (29%)	0	37 (90%)
Rash^b^^,^^c^	16 (39%)	0	36 (88%)
Nausea	1 (2%)	0	34 (83%)
Neutropenia or neutrophil count decreased^c^^,^^d^	21 (51%)	4 (10%)	32 (78%)
Fatigue	4 (10%)	0	30 (73%)
Constipation	0	0	27 (66%)
Diarrhea	3 (7%)	0	24 (59%)
Vomiting	0	0	24 (59%)
Dysgeusia	0	0	21 (51%)
Upper respiratory tract infection	0	0	21 (51%)
Insomnia	1 (2%)	0	19 (46%)
Headache	0	0	18 (44%)
Anemia or hemoglobin decrease^c^	5 (12%)	0	17 (42%)
Pruritus	4 (10%)	0	17 (42%)
Urinary tract infection	1 (2%)	1 (2%)	17 (42%)
White blood cell count decreased or leukopenia^c^	8 (20%)	1 (2%)	17 (42%)
Arthralgia	0	0	16 (39%)
Back pain	0	0	16 (39%)
Hyperglycemia or blood glucose increased^c^	3 (7%)	0	14 (34%)
Oropharyngeal pain	0	0	14 (34%)
Pyrexia	1 (2%)	0	14 (34%)
AST or ALT increased^c^	3 (7%)	0	13 (32%)
Dizziness	0	0	13 (32%)
Cough	0	0	12 (29%)
Epistaxis	0	0	12 (29%)
Hot flush	0	0	12 (29%)
Hypokalemia	2 (5%)	0	12 (29%)
Influenza-like illness	0	0	12 (29%)
Infusion-related reaction	0	0	12 (29%)
Dry mouth	0	0	11 (27%)
Hypomagnesemia	0	0	11 (27%)
Decreased appetite	0	0	10 (24%)
Dry skin	0	0	10 (24%)
Hypertension	4 (10%)	0	10 (24%)
Lymphocyte count decreased	4 (10%)	0	9 (22%)
Myalgia	0	0	9 (22%)
Nasal congestion	0	0	9 (22%)
Oral pain	0	0	9 (22%)

Abbreviations: ALT, alanine aminotransferase; AST, aspartate aminotransferase.

a Prophylactic treatment for stomatitis was not implemented.

b Includes rash, rash maculopapular, rash pruritic, rash papular, rash erythematous, dermatitis, and dermatitis acneiform.

c Number of patients with at least one of the terms. If a patient experienced multiple terms, it was counted once for the highest grade.

d Neutropenia and neutrophil count decrease were reported interchangeably for many patients. In this table, neutropenia (system organ class blood and lymphatic system disorders) and neutrophil count decreased (system organ class investigations) were combined.

**Table 4.** Grade 3 or 4 TESAEs, of any cause, occurring in >1 patient, *N* = 41.

Preferred term	Grade 3 *N* (%)	Grade 4 *N* (%)
Patients with at least one TESAE	15 (37%)	3 (7%)
Febrile neutropenia	5 (12%)	0
Acute kidney injury	2 (5%)	0
Bacteremia	1 (2%)	1 (2%)
Stomatitis or mucosal inflammation	2 (5%)	0
Urinary tract infection	1 (2%)	1 (2%)
Hypercalcemia	0	1 (2%)
Sepsis	0	1 (2%)

Number of patients with at least one of the terms. If a patient experienced multiple terms, it was counted once for the highest grade. All Grade 4 TESAEs are presented. Two grade 4 TESAEs occurred in one patient (grade 4 urinary tract infection and grade 4 bacteremia).

## Discussion

In treatment-naïve patients with HR+, HER2– advanced breast cancer, gedatolisib in combination with palbociclib and letrozole induced an ORR of 79% among evaluable patients, a median PFS of 48.4 months, and a median OS of 77.3 months. The triplet therapy had a low rate of discontinuations due to treatment-related AEs [<10% (4/41)], and side effects were managed by available standards of care. The efficacy and safety results of the pooled analysis were consistent with the study results published previously for the dose expansion arm A patients (11).

A subgroup analysis of PFS and ORR by *PIK3CA* status found that these results were comparable in patients with and without *PIK3CA* mutations, which is consistent with results reported previously for the expansion arms of the study (11). The comparable activity of the triplet combination in patients with and without *PIK3CA* mutations may, in part, be explained by the unique mechanism of action of gedatolisib. Gedatolisib targets all class I PI3K isoforms and mTORC1 and mTORC2 to induce comprehensive inhibition of the PAM pathway (14). This contrasts with the currently approved inhibitors targeting single nodes of the PAM pathway (15). Alpelisib is a p110α subunit–selective PI3K inhibitor (16), inavolisib is a highly selective PI3Kα inhibitor that also promotes the degradation of the mutant p110α isoform (17), everolimus is an mTORC1 inhibitor (18), and capivasertib is an AKT inhibitor (19). The multinode inhibition exhibited by gedatolisib not only may prevent compensatory feedback and cross-talk between the different pathway nodes but may also elicit a response in a broader patient population as the mechanism of action does not depend on a specific mutation subtype.

When combined with letrozole, the three approved CDK4/6 inhibitors—palbociclib, ribociclib, and abemaciclib—have each reported significant improvements in PFS compared with letrozole alone as first-line treatment for women with HR+, HER2− advanced breast cancer whose disease recurred more than 12 months after completing adjuvant ET. The PALOMA-2 study evaluated palbociclib combined with letrozole as first-line therapy for patients with HR+, HER2− advanced breast cancer, a similar patient population to the subgroup analyzed here, which thus provides a baseline measure of efficacy for one of the current standard-of-care regimens (20, 21). For patients treated with palbociclib and letrozole in PALOMA-2, the median PFS was 27.6 months, and the ORR was 55% (22). Similar studies with ribociclib (MONALEESA-2) and abemaciclib (MONARCH 3; combined with a nonsteroidal aromatase inhibitor), both of which were combined with letrozole as initial therapy for advanced breast cancer, reported median PFS of 25.3 and 28.2 months, respectively (22–24). Although cross-trial analyses should be approached with caution, comparing the results from this study with those from the phase III PALOMA-2 study is informative. Patients enrolled in the PALOMA-2 trial included 48% with visceral disease, 23% with bone-only disease, and 76% with measurable disease. By contrast, the patient population analyzed here included 78% with visceral metastasis, only 1 (2%) with bone-only disease, and 93% with measurable baseline disease and reported median PFS of 48.4 months and ORR of 79%.

A patient’s endocrine resistance or sensitivity status has recently been incorporated into guidelines recommending first-line treatment options (25, 26). Although the current study was designed prior to the adoption of these guidelines, the patients enrolled would be classified as ET-sensitive as their disease recurrence occurred more than 12 months after completing their adjuvant ET. Primary and secondary endocrine resistances (defined according to time to relapse from adjuvant ET) are associated with worse survival, with an adjusted HR for death of 1.54 in patients with primary endocrine resistance and 1.17 in patients with secondary endocrine resistance, compared with patients with endocrine-sensitive disease (27). Patients with ET-resistant, *PIK3CA*-mutated, HR+/HER2− locally advanced or metastatic treatment-naïve breast cancer were recently evaluated in the INAVO120 phase III clinical trial that compared inavolisib, a PI3Kα inhibitor, in combination with palbociclib and fulvestrant with standard-of-care palbociclib plus fulvestrant. Median PFS was 15.0 months (95% CI, 11.3–20.5) in the group receiving inavolisib plus palbociclib and fulvestrant and 7.3 months (95% CI, 5.6–9.3) in the placebo plus palbociclib and fulvestrant group. Based on these results, the FDA recently approved inavolisib, a PI3Kα inhibitor, when combined with palbociclib and fulvestrant to treat patients with endocrine-resistant HR+/HER2− advanced breast cancer. Further, based on clinicaltrials.gov, the INAVO123 study is planned to evaluate inavolisib combined with letrozole and a CDK4/6 inhibitor in endocrine-sensitive breast cancer (NCT06790693). Unlike inavolisib, which is only indicated for patients with *PIK3CA*-mutated breast cancer, gedatolisib has demonstrated activity in patients with or without *PIK3CA* mutations. These results provide further evidence of the opportunity to improve clinical outcomes in the first-line setting by adding a PAM inhibitor to a standard-of-care CDK4/6 plus ET regimen.

The limitations associated with this subpopulation analysis report include its small sample size and the *post hoc* analysis from two different arms of the trial. In particular, the response evaluation by *PIK3CA* mutation status involved small patient numbers and was an exploratory and hypothesis-generating analysis. Although most patients included in the analysis were assigned to a predefined cohort based on prior treatment, we also included patients from the dose escalation portion of the study who met the same prior treatment criteria. However, the patient inclusion criteria in the dose escalation portion did not require measurable baseline disease. Pooling these patients for analysis provided a larger study population from which to draw preliminary conclusions. Although the potential benefit of treating patients with a gedatolisib triplet regimen in the first-line setting is compelling, it must be noted that the intravenous administration of gedatolisib increases patient burden relative to current standard-of-care regimens. There is currently a lack of safety data for a triplet regimen with gedatolisib, ET, and other current kinase inhibitors such as ribociclib or abemaciclib although a phase III study including gedatolisib in combination with ribociclib or palbociclib is underway (NCT06757634). Furthermore, treatment with a gedatolisib triplet regimen may add toxicity better justified in a first-line endocrine-resistant or later-line setting, highlighting the importance of a careful assessment of the benefit/risk profile of this triplet regimen. Nonetheless, it is encouraging that in this preliminary study of the gedatolisib triplet regimen, the discontinuation rate of study treatment due to AEs was comparable with the discontinuation rate observed in the PALOMA-2 study that evaluated palbociclib and letrozole.

In conclusion, the ORR, median PFS, and OS for patients who received gedatolisib combined with palbociclib and letrozole compare favorably with published results for patients receiving palbociclib and letrozole as first-line therapy for advanced breast cancer. Additionally, the proportion of patients discontinuing treatment due to treatment-related AEs in this study and for palbociclib and letrozole was identical (∼10%). In light of these promising preliminary efficacy and safety results, a phase III study evaluating gedatolisib combined with a CDK4/6 inhibitor and fulvestrant as first-line treatment for patients with ET-resistant HR+, HER2− advanced breast cancer was initiated (NCT06757634; VIKTORIA-2). VIKTORIA-2 will enroll patients whose disease progressed during or within 12 months of completing neoadjuvant or adjuvant ET. An additional phase III study evaluating gedatolisib combined with palbociclib and fulvestrant in patients with HR+/HER2− advanced breast cancer that progressed after treatment with a CDK4/6 inhibitor and an aromatase inhibitor is also underway (NCT05501886).

## Supplementary Material

Supplementary Appendix1 Table S1 and Figure S1

## References

[1] Waks AG , Winer EP. Breast cancer treatment: a review. JAMA 2019;321:288–300. 30667505 10.1001/jama.2018.19323

[2] Jerzak KJ , Bouganim N, Brezden-Masley C, Edwards S, Gelmon K, Henning JW, . HR+/HER2− advanced breast cancer treatment in the first-line setting: expert review. Curr Oncol 2023;30:5425–47. 37366894 10.3390/curroncol30060411 PMC10297170

[3] Lambertini M , Blondeaux E, Bisagni G, Mura S, De Placido S, De Laurentiis M, . Prognostic and clinical impact of the endocrine resistance/sensitivity classification according to international consensus guidelines for advanced breast cancer: an individual patient-level analysis from the Mammella InterGruppo (MIG) and Gruppo Italiano Mammella (GIM) studies. eClinicalMedicine 2023;59:101931. 37256095 10.1016/j.eclinm.2023.101931 PMC10225659

[4] Cardoso F , Paluch-Shimon S, Senkus E, Curigliano G, Aapro MS, André F, . 5th ESO-ESMO international consensus guidelines for advanced breast cancer (ABC 5). Ann Oncol 2020;31:1623–49. 32979513 10.1016/j.annonc.2020.09.010 PMC7510449

[5] Alves CL , Ditzel HJ. Drugging the PI3K/AKT/mTOR pathway in ER+ breast cancer. Int J Mol Sci 2023;24:4522. 36901954 10.3390/ijms24054522 PMC10003259

[6] Clark AS , Makhlin I, DeMichele A. Setting the pick: can PI3K inhibitors circumvent CDK4/6 inhibitor resistance? Clin Cancer Res 2021;27:371–3. 33144339 10.1158/1078-0432.CCR-20-3624 PMC8278622

[7] Garrido-Castro AC , Goel S. CDK4/6 inhibition in breast cancer: mechanisms of response and treatment failure. Curr Breast Cancer Rep 2017;9:26–33. 28479958 10.1007/s12609-017-0232-0 PMC5414585

[8] Vasan N , Toska E, Scaltriti M. Overview of the relevance of PI3K pathway in HR-positive breast cancer. Ann Oncol 2019;30(Suppl 10):x3–11. 31859348 10.1093/annonc/mdz281 PMC6923788

[9] O’Brien NA , McDermott MSJ, Conklin D, Luo T, Ayala R, Salgar S, . Targeting activated PI3K/mTOR signaling overcomes acquired resistance to CDK4/6-based therapies in preclinical models of hormone receptor-positive breast cancer. Breast Cancer Res 2020;22:89. 32795346 10.1186/s13058-020-01320-8 PMC7427086

[10] Turner NC , Im SA, Saura C, Juric D, Loibl S, Kalinsky K, . Inavolisib-based therapy in PIK3CA-mutated advanced breast cancer. N Engl J Med 2024;391:1584–96. 39476340 10.1056/NEJMoa2404625

[11] Layman RM , Han HS, Rugo HS, Stringer-Reasor EM, Specht JM, Dees EC, . Gedatolisib in combination with palbociclib and endocrine therapy in women with hormone receptor-positive, HER2-negative advanced breast cancer: results from the dose expansion groups of an open-label, phase 1b study. Lancet Oncol 2024;25:474–87. 38547892 10.1016/S1470-2045(24)00034-2

[12] Diehl F , Li M, Dressman D, He Y, Shen D, Szabo S, . Detection and quantification of mutations in the plasma of patients with colorectal tumors. Proc Natl Acad Sci U S A 2005;102:16368–73. 16258065 10.1073/pnas.0507904102 PMC1283450

[13] Dressman D , Yan H, Traverso G, Kinzler KW, Vogelstein B. Transforming single DNA molecules into fluorescent magnetic particles for detection and enumeration of genetic variations. Proc Natl Acad Sci U S A 2003;100:8817–22. 12857956 10.1073/pnas.1133470100 PMC166396

[14] Gazi M , Moharram SA, Marhäll A, Kazi JU. The dual specificity PI3K/mTOR inhibitor PKI-587 displays efficacy against T-cell acute lymphoblastic leukemia (T-ALL). Cancer Lett 2017;392:9–16. 28159681 10.1016/j.canlet.2017.01.035

[15] Sarbassov DD , Ali SM, Kim DH, Guertin DA, Latek RR, Erdjument-Bromage H, . Rictor, a novel binding partner of mTOR, defines a rapamycin-insensitive and raptor-independent pathway that regulates the cytoskeleton. Curr Biol 2004;14:1296–302. 15268862 10.1016/j.cub.2004.06.054

[16] Juric D , Janku F, Rodón J, Burris HA, Mayer IA, Schuler M, . Alpelisib plus fulvestrant in PIK3CA-altered and PIK3CA-wild-type estrogen receptor-positive advanced breast cancer: a phase 1b clinical trial. JAMA Oncol 2019;5:e184475. 30543347 10.1001/jamaoncol.2018.4475 PMC6439561

[17] Hanan EJ , Braun MG, Heald RA, MacLeod C, Chan C, Clausen S, . Discovery of GDC-0077 (inavolisib), a highly selective inhibitor and degrader of mutant PI3Kα. J Med Chem 2022;65:16589–621. 36455032 10.1021/acs.jmedchem.2c01422

[18] Schuler W , Sedrani R, Cottens S, Häberlin B, Schulz M, Schuurman HJ, . SDZ RAD, a new rapamycin derivative: pharmacological properties in vitro and in vivo. Transplantation 1997;64:36–42. 9233698 10.1097/00007890-199707150-00008

[19] Turner NC , Oliveira M, Howell SJ, Dalenc F, Cortes J, Gomez Moreno HL, . Capivasertib in hormone receptor-positive advanced breast cancer. N Engl J Med 2023;388:2058–70. 37256976 10.1056/NEJMoa2214131 PMC11335038

[20] IBRANCE [prescribing information]. Pfizer Inc.; 2023 [cited 2024 May 21].

[21] Finn RS , Martin M, Rugo HS, Jones S, Im SA, Gelmon K, . Palbociclib and letrozole in advanced breast cancer. N Engl J Med 2016;375:1925–36. 27959613 10.1056/NEJMoa1607303

[22] Rugo HS , Finn RS, Diéras V, Ettl J, Lipatov O, Joy AA, . Palbociclib plus letrozole as first-line therapy in estrogen receptor-positive/human epidermal growth factor receptor 2-negative advanced breast cancer with extended follow-up. Breast Cancer Res Treat 2019;174:719–29. 30632023 10.1007/s10549-018-05125-4 PMC6438948

[23] Hortobagyi GN , Stemmer SM, Burris HA, Yap YS, Sonke GS, Paluch-Shimon S, . Updated results from MONALEESA-2, a phase III trial of first-line ribociclib plus letrozole versus placebo plus letrozole in hormone receptor-positive, HER2-negative advanced breast cancer. Ann Oncol 2019;30:1842. 31407010 10.1093/annonc/mdz215 PMC6927326

[24] VERZENIO® (abemaciclib) tablets, for oral use prescribing information. Eli Lilly and Company; 2023 [cited 2024 Oct 6]. Available from: https://uspl.lilly.com/verzenio/verzenio.html#pi.

[25] Burstein HJ , Somerfield MR, Barton DL, Dorris A, Fallowfield LJ, Jain D, . Endocrine treatment and targeted therapy for hormone receptor-positive, human epidermal growth factor receptor 2-negative metastatic breast cancer: ASCO guideline update. J Clin Oncol 2021;39:3959–77. 34324367 10.1200/JCO.21.01392 PMC8659999

[26] Gennari A , André F, Barrios CH, Cortés J, de Azambuja E, DeMichele A, . ESMO Clinical Practice Guideline for the diagnosis, staging and treatment of patients with metastatic breast cancer. Ann Oncol 2021;32:1475–95. 34678411 10.1016/j.annonc.2021.09.019

[27] Lambertini M , Blondeaux E, Bisagni G, Mura S, De Placido S, De Laurentiis M, . Prognostic and clinical impact of the endocrine resistance/sensitivity classification according to international consensus guidelines for advanced breast cancer: an individual patient-level analysis from the Mammella InterGruppo (MIG) and Gruppo Italiano Mammella (GIM) studies. EClinicalMedicine 2023;59:101931. 37256095 10.1016/j.eclinm.2023.101931 PMC10225659

