# Supplementary material for: Gedatolisib Combined with Palbociclib and Letrozole in Patients with No Prior Systemic Therapy for Hormone Receptor–Positive, HER2-Negative Advanced Breast Cancer
Source: Clin Cancer Res. 2025 Jul 25;31(19):4040–8. doi: 10.1158/1078-0432.CCR-25-0992 (PMC12485373; doi:10.1158/1078-0432.CCR-25-0992)
Supplement: Supplementary Appendix1 — Table S1 and Figure S1 [file ccr-25-0992_supplementary_appendix1_suppas1.docx]

**Supplemental Appendix**

Supplementary Table 1. Representativeness of Study Participants

| Cancer type | HR+, HER2- advanced breast cancer (ABC) |
| --- | --- |
| Considerations related to: |  |
| Sex | In the USA, HR+, HER2- ABC, similar to all breast cancer subtypes, is predominantly a female disease and is rare in men. Male breast cancers represent 1% of breast cancer overall and <1% of all male tumors. |
| Age | In the USA, median age of all breast cancer at diagnosis of all races/ethnicities is around 63 years. In HR+, HER2- ABC, the median age is around 64 years. |
| Race/Ethnicity | In the USA from 2017 to 2021, the HR+, HER2- breast cancer incidence per 100,000 cases was 76.1 among black women, 77.5 among Asian/Pacific Islander, 77.5 among American Indian/Alaska native, 100.8 cases among white women and 66.3 among Hispanic women (any race). |
| Geography | In the US, HR+, HER2- breast cancer prevalence is about 70% of all female breast cancer subtypes according to SEER 22 (data from 2017-2021), with an incidence of 90 per 100,000 US women. |
| Other considerations | In a study evaluating racial and ethnic representation in US oncology trials, among the cohorts evaluating treatments for metastatic breast cancer, the percentage of black population was 15.2, the percentage of LatinX population was 10.6, the percentage of white participants was 74.3.  The 5-year survival rate (2016 SEER data) of HR+, HER2- breast cancer is 95% overall, 89.8% among black women and 95.6% among white women. |
| Overall representativeness of this study | The age distribution of our study analysis is a median age of 54 years. No men were recruited for this study. Black women represented 12% of the study analysis, white women 85%, and other races/ethnicities 2%. All study sites were in the USA. |

SEER*Explorer: An interactive website for SEER cancer statistics [Internet]. Surveillance Research Program, National Cancer Institute; 2024 Apr 17. [updated: 2024 Nov 5; cited 2025 Mar 22]. Available from: https://seer.cancer.gov/statistics-network/explorer/. Data source(s): SEER Incidence Data, November 2023 Submission (1975-2021), SEER 22 registries (excluding Illinois and Massachusetts). Expected Survival Life Tables by Socio-Economic Standards.

JAMA Network Open. 2023;6(7):e2322515. doi:10.1001/jamanetworkopen.2023.22515

Figure S1. Patient flow diagram


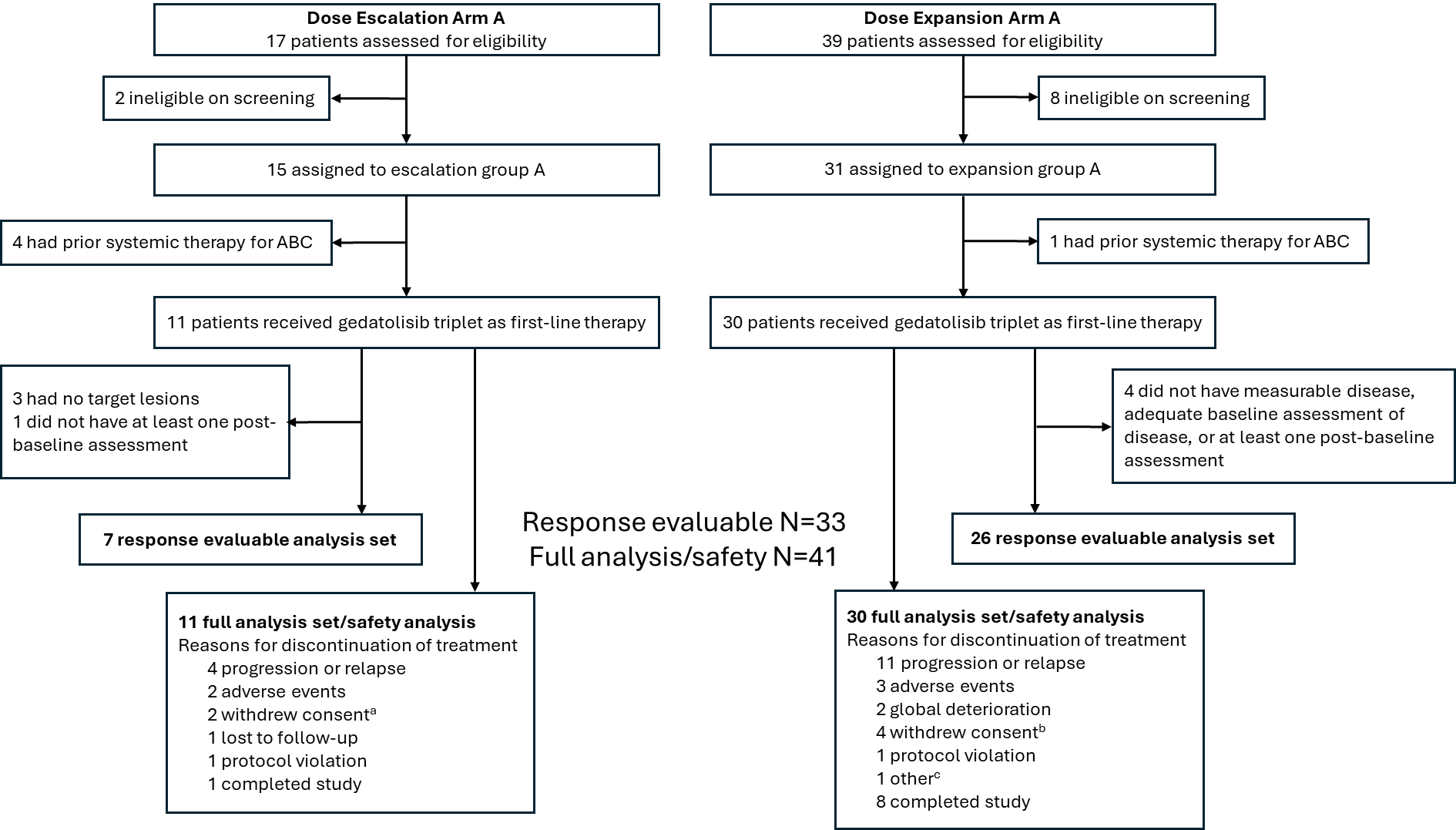


^a^ reason specified for withdraw of consent: unknown (n=1), refusal of traditional therapy for holistic medicine (n=1)

^b^ reason specified for withdraw of consent: unknown (n=3), general side effects (n=1)

^c^ patient discontinued due to undergoing treatment for other malignancy

All subjects who “completed study” continued treatment in an expanded access protocol or in a single patient Initial New Drug Application (IND)
